# Advancements in Inactivation of Soybean Trypsin Inhibitors

**DOI:** 10.3390/foods14060975

**Published:** 2025-03-12

**Authors:** Zhanjun Luo, Yujia Zhu, Huiyu Xiang, Ziqian Wang, Zhimo Jiang, Xinglong Zhao, Xiaomeng Sun, Zengwang Guo

**Affiliations:** 1 College of Food Science, Northeast Agricultural University, Harbin 150030, China; 18571677079@163.com (Z.L.); 13359603256@163.com (Y.Z.); 13534253985@163.com (H.X.); 18245114962@163.com (Z.W.); 17597366749@163.com (Z.J.); 2 College of Engineering, Northeast Agricultural University, Harbin 150030, China; zhaoxinglong@neau.edu.cn; 3 Center for Innovation and Entrepreneurship, Northeast Agricultural University, Harbin 150030, China

**Keywords:** soybean trypsin inhibitors, KTI, BBI, physical treatments, thermal, chemical, inactivation

## Abstract

Soybean Trypsin Inhibitors (STIs) in soy-based foods have negative effects on soybean protein digestion and pancreatic health of humans. The inactivation of STIs is a critical unit operation aimed at enhancing the nutritional properties of soy-based foods during processing. This paper reviews the structure of STIs and soybean proteins, as well as the mechanisms of digestion. Various technologies (physical, chemical, biological) have been used to inactivate STIs. Their parameter settings, operating procedures, advantages, and disadvantages are also described. Mechanisms of inactivation of STIs (Kunitz trypsin inhibitor (KTI) and Bowman–Birk inhibitor (BBI)) conformations under different treatments are clarified. In addition, emerging technologies, e.g., Ohmic Heating, Electron Beam Irradiation, Dielectric-Barrier Discharge, and probiotics, have demonstrated great potential to inactivate STIs. We advise that multiple emerging technologies should combine with other unit operating systems to maximize inactivation efficiency.

## 1. Introduction

The global soybean planting area and annual production have been steadily increasing as the demand for feed proteins and vegetable oils rises rapidly. Soybeans planted in Latin America account for 85.7% of the global total yield. The total soybean yield in 2022 is 50% higher than that in 2019 (All the above data are from the Food and Agriculture Organization of the United Nations (FAO). https://www.fao.org). As an indispensable crop, soybean can provide not only sufficient nutrients, e.g., amino acids, proteins, minerals, and vitamins, but also many bioactive compounds, e.g., soybean isoflavones, saponins, and phenolic compounds with antioxidant, anti-pathogen, anti-inflammatory, and anti-cancer [1]. Due to excellent physicochemical and functional properties, soybean protein finds extensive application in various fields, e.g., fat replacer [2], ice cream [3], plant-based dressings [4], soymilk [5], encapsulation carriers of bioactive substances [6], 3D food printing [7], yogurt [8], plant-based meat [9], and food cling film [10].

**Figure 1:**
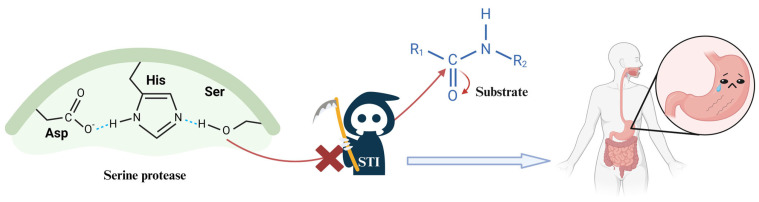
Mechanism of Soybean Trypsin Inhibitors affecting the digestive property of trypsin.

Though soybean has nutritional characteristics, it also contains many anti-nutritional bioactive substances (Table S1) that have negative effects on the metabolic and physiological activities of human including Lipoxygenase (LOX), urease, Soybean Trypsin Inhibitors (STIs), Soybean agglutinin (SBA), Tannic acid, Phytic acid, Urease, Saponin, and Isoflavones. Most of the antinutritional factors (ANFs) are derived from whey soy protein, which included STIs in the 2S component and SBA, LOX classified as 7S [11,12]. These antinutritional factors can restrict the digestion and absorption of certain nutrients. As an illustration, the over-secreted mucus stimulated by SBA can affect the enzymatic reactions and absorptive capacity of the intestinal wall [4]. The lipoxygenase enzyme can catalyze the oxidation of polyunsaturated fatty acids, like linoleic and linolenic acid, resulting in unpleasant flavor [13,14]. STIs form complexes with trypsin/serine proteases to inhibit their activity and are widely found in animals, plants, and microbes [15]. In some commercial soybean products, urease activity (by the American Oil Chemists’ Society (AOCS) official method) can be indirectly used to assess the inactivation of STIs. The two main STIs—Kunitz trypsin inhibitor (KTI) and Bowman–Birk inhibitor (BBI)—draw attention due to their 10% share in seed protein and significant impact on digestion (Figure 1) [16]. Studies show that STIs in soybean products can exceed 30 mg/g, leading to much lower digestibility of soybean protein (66.1%) than that of whey protein (75.5%) [17]. While other ANFs in soybeans affect nutrient absorption in humans, their impact is less significant and widespread compared to STIs. This study focuses on STIs, particularly KTI and BBI, emphasizing their method and mechanism of inactivation.

**Figure 2:**
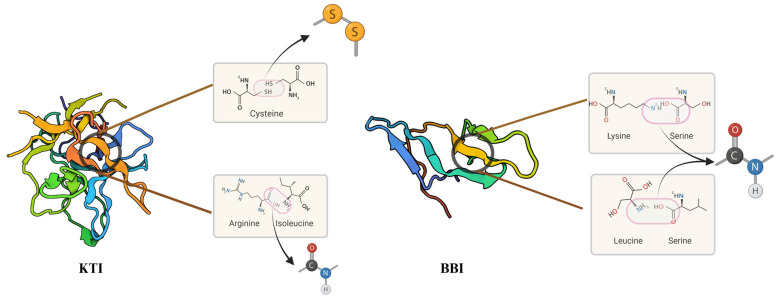
Two configurations of Soybean Trypsin Inhibitors (Kunitz trypsin inhibitor (KTI) and Bowman–Birk inhibitor (BBI)).

KTI protein is a non-glycosylated, monomeric, and globulin-type protein with a molecular weight of about 21.5 kDa and an isoelectric point of pH 4.5 [18]. It consists of approximately 170 to 200 amino acid residues, which contain two disulfide bonds, including Cys 138-Cys 145 and Cys 39-Cys 86 (Figure 2). KTI also has 12 crisscrossed antiparallel β−strands linked to each other by hydrogen bonds, forming a stable protein structure [18,19]. The active centers of KTI are situated at arginine (Arg) at position 63 and isoleucine (Ile) at position 64; they can react with trypsin at the ratio of 1:1 to inactivate it.

BBI is a protein of 71 amino acids cross-linked by seven disulfide bonds. As shown in Figure 2, BBI has two independent sites of inhibition, one at lysine (Lys) 16-serine (Ser) 17 against trypsin, and the other at Leucine (Leu) 43-Ser 44 against chymotrypsin. The BBI exhibits a dual inhibitory mechanism through distinct structural domains: Trypsin inhibition: The N-terminal domain contains Lys16, which forms hydrogen bonds with Asp189, directly obstructing the enzyme’s catalytic site. Chymotrypsin/elastase inhibition: The C-terminal domain utilizes the hydrophobic side chain of Leu43 to bind the hydrophobic pocket of chymotrypsin, thereby suppressing both chymotrypsin and elastase activities [20]. Both sites consist of two symmetrically relevant β-folded lamellae with each containing antiparallel β-chains. It can form a binary complex with either trypsin or chymotrypsin at the ratio of 1:1 and a ternary complex with both two enzymes [21]. BBI has higher thermal stability than KTI. The trypsin inhibitor activity (TIA) of KTI in soymilk can be completely inactivated by heating for 20 min in boiling water, while only approximately 5% of chymotrypsin inhibitor activity (CIA) of BBI can be inactivated (KTI acts on trypsin, while BBI acts on trypsin and chymotrypsin) [22,23]. During heating, KTI can aggregate with other proteins either through non-covalent interactions or via disulfide bonding, which results in a decrease in TIA. Furthermore, the TIA of free KTI may be decreased due to structural alterations. In contrast, BBI retains structural integrity, suggesting that any residual TIA and CIA in heated soymilk are likely attributed to BBI [24].

Regulatory agencies do not require limits on STIs in soy products due to their low-risk profile. Moreover, digestion also can reduce the bioactivities of STIs. Therefore, STIs have little effect on health after being processed. In contrast, STIs could also be applied in the prevention of cancer, bacterial infection, Dengue fever, inflammatory, and allergic disorders, due to their serine protease activity [25,26,27,28,29,30]. The effects of STIs on humans and animals are significantly different. STIs exhibit dose-dependent effects in animals [31]. Their anti-nutritional effects manifest as suppressed intestinal proteolytic enzyme activity, reduced digestibility of amino acids (AAs) and minerals, and increased endogenous Amino acid losses [32]. Purified STIs increased absolute and relative pancreatic weights, particularly at 14–21 days post-hatching in bird studies [33]. STIs form irreversible complexes with trypsin in the small intestine, inhibiting enzyme function and triggering compensatory hypersecretion of digestive enzymes, leading to pancreatic hypertrophy [34]. Although soybean lectins may influence pancreatic function by polyamine accumulation, they have less impact on the pancreas [35], confirming STIs as the primary ANFs in animals. In summary, the dose-response mechanisms of STIs in animals have been well-characterized, but their intake levels, metabolic pathways, and safety thresholds in humans have significant differences due to distinct physiological environments.

Due to the distinct three-dimensional architectures and active sites composed of disulfide bonds, as well as various thermal properties of STIs, thermal processing is a commonly used inactivation of KTI. While reducing agents and enzymatic treatments are considered more suitable methods for BBI. Thermal treatments are the traditional methods for STIs inactivation used in industry, which are time-consuming and inefficient. The novel technologies, e.g., Ohmic Heating, Pulsed Electric Field, Dielectric-Barrier Discharge, and fermentation, have proven the ability to inactivate STIs and improve the structure and functionality of plant proteins [7]. These technologies have advantages in food safety and energy efficiency, which attracts more and more attention in the food scientific field. Table 1, Table 2 and Table 3 list popular treatments (physical, chemical, biological) and describe the parameters, advantages, and disadvantages of each treatment.

This review aimed primarily to introduce the types of soybean STIs and explain the mechanisms of how STIs affect the digestion and absorption of soy proteins. It also highlighted the physical treatments, chemical treatments, and biological treatments to inactivate STIs and relevant operation processes, advantages, and disadvantages. In particular, co-processing is illustrated as a future trending treatment. Kunitz trypsin inhibitor (KTI) and Bowman–Birk inhibitor (BBI) are the two main configurations of STIs. Previous studies have discussed the inactivation of KTI [17,60]. BBI has not been extensively studied. We will discuss the mechanism of STIs inactivation comprehensively by two configurations of KTI and BBI. Our article provides practical significance for the research and development of more nutritious soy-based foods.

## 2. Soybean Proteins and Their Digestion

### 2.1. Soybean Proteins

Soybean proteins are recognized as high-quality proteins (average DIAAS of ≥75) by the Digestible Indispensable Amino Acid Score (DIAAS) [61]. They can be classified as salt-soluble globulins, water-soluble albumins, dilute acid/alkali-soluble glutelins, and alcohol-soluble prolamins [62] mainly originating from nitrogen fixation by soybean root nodule bacteria [63]. Globulins, the major component of soybean proteins (80–90%), can be further categorized into 11S globulin (also known as glycinin) and 7S globulin (also known as β-conglycinin, β-CG) based on their sedimentation coefficients [64,65].

β-CG is a 7S trimer glycoprotein with a molecular weight around 180 kDa. It is composed of three glycosylation subunits, (α, α, and β) that can form three homo-trimers and seven hetero-trimers through random hydrophobic and electrostatic interactions [66]. The β-CG secondary structure is characterized by a notable presence of α-helices and β-sheets, with some variations observed among different soybean varieties. Furthermore, the β-CG structure’s antigenic and allergenic particular areas are related to soybean protein allergies, which can cause immune system reactions in people with sensitivity [67]. The 11S fraction predominantly contains soybean glycinin (molecular weight 300–380 kDa), which makes up over 30% of the total protein content in soybeans. It is a hexamer consisting of five major subunits. Each subunit is composed of an acidic polypeptide chain and an alkaline polypeptide chain connected by disulfide bonds. Two trimers formed by acidic-basic peptide pairs via hydrophobic and/or hydrogen bonding forces can form the glycinin hexamer with the position one on the top of the other [66,68].

### 2.2. The Digestion of Soybean Proteins

Soybean proteins in soybean have comparable content with milk, meat, and eggs according to the Protein Digestibility Corrected Amino Acid Score (PDCAAS) [69]. However, compared to proteins of animal origin (90–95%), plant proteins usually have lower digestibility (around 75–80%) [70]. The reason is that soybean protein is usually encapsulated by cellulose, which limits the contact between digestive enzymes and protein [71]. During gastrointestinal digestion, soybean proteins may be degraded into various small molecular bioactive peptides. In contrast to 11S, 7S is more resistant to trypsin/chymotrypsin proteolysis in the intestines because of its ability to form amyloid aggregates [72,73]. Moreover, the 7S protein demonstrates greater susceptibility to oxidative modifications compared to 11S, with the β subunit of β-CG also displaying higher resistance to digestion following oxidative modification [74].

### 2.3. The Changes and Effects of STIs in Gastrointestinal Tract

STIs have different changes in the stomach and small intestine. The activity of STIs usually decreases by 30–40% when exposed to human gastric juice (pH = 1.5–3.5) [75]. KTI can be rapidly inactivated following the disruption of structural integrity, while BBI has greater stability. STIs can prolong the retention time of food in the gastrointestinal tract especially in the small intestine [76]. Because the pancreas is adjacent to the duodenum, it is easier for STIs to form complexes with trypsin and decrease the activity of trypsin.

The interaction between STIs and trypsin is a complicated process. At a fundamental level, STIs bind the active site of trypsin specifically by hydrogen bonds, hydrophobic interactions, electrostatic interactions, and van der Waals forces [77]. Especially, the salt bridge is formed by the Lys or Arg residues of STIs with the aspartate (Asp) of trypsin. It is crucial in the specific recognition and binding of STIs [78]. Furthermore, hydrophobicity contributes to STIs-trypsin complex stability by reducing the solvent-accessible surface area to increase solvent entropy [79]. During the binding process, the secondary structure of trypsin is changed (decrease in α-helical and increase in β-sheet), which may be associated with protein aggregation and fibrillation [80], and the disulfide bonds may undergo rearrangement which causes the exchanged Cys 14-Cys 38 in TIs with Cys 179-Cys 203 or Cys 31-Cys 47 in trypsin. The rearrangement also forms more stable covalent bonds [7] and affects the stability and kinetic properties of the complex [81].

The interaction between KTI and trypsin predominantly results in the specific binding loop of KTI contacts with the active site of trypsin. This binding mode is the precise alignment of the reactive site of KTI with key residues of trypsin (Ser 195, Histidine (His) 57, and Asp 102), leading to changes in trypsin structure and inhibition of activity [82]. While BBI has dual inhibitory effects by engaging with trypsin at two distinct binding sites [83]. BBI forms a non-covalent complex with chymotrypsin and trypsin to inactivate it. Specifically, the exposed hydrophobic and polar regions in BBI’s structure form complementary interactions with the active site of chymotrypsin and trypsin, leading to the inhibition effect [20].

## 3. Inactivation Technologies for STIs

### 3.1. Conventional Treatments

#### 3.1.1. Thermal Treatments

**Figure 3:**
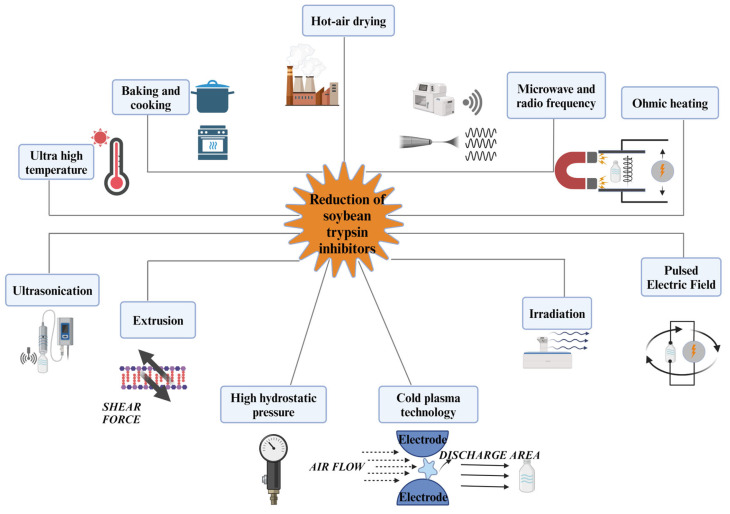
The physical treatments to inactivate Soybean Trypsin Inhibitors, including conventional thermal treatments, emerging thermal treatments, and combined temperature–pressure treatments, etc.

**Figure 4:**
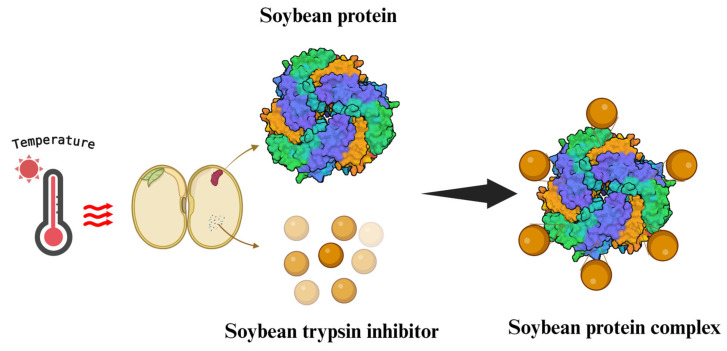
Mechanism of Soybean Trypsin Inhibitors (mostly Kunitz trypsin inhibitor) inactivation under thermal treatment.

Figure 3 demonstrates prevalent physical treatments. A variety of thermal treatments, including radio frequency, infrared heating, and conventional heating (baking, boiling, blanching, and drying) commonly are applied in soybean processing to reduce the activity of STIs [22]. According to reports, heating at 200 °C for 20 min in an oven could significantly reduce the STIs in whole soybean flour [37]. Under high temperatures (>95 °C), the quaternary structure and sugar-binding sites of SBA can be disrupted, and its activity can be significantly reduced [84]. Studies have shown that the ideal inactivation of trypsin inhibitor and lectin can be achieved without greatly lowering protein solubility at 121 °C for 15 min [85]. The fluidized bed drying with the inlet air temperature from 80 °C to 60 °C for 30 min could also reduce 86% STIs in soybean flour [40]. About 64 and 69% of TIA in soy-based foods could be decreased by hot-grinding compared with cold and ambient grinding. Moreover, TIA almost disappeared, and the CIA was decreased by 33% after boiling for 20 min as reported by Zhang and Chang [36]. The reason was that the lipid hydroperoxides (produced by oxidation of unsaturated fatty acids in soybeans) in soybean flour oxidized the disulfide bonds of STIs and generated monoxide or dioxide [86]. In the heating-soymilk system, KTI existed independently or incorporated into small aggregates by disulfide or noncovalent bonds [87]. The active sites of KTIs could be buried inside to reduce the possibility of STIs binding with trypsin (Figure 4). In contrast, BBI is not inclined to form intermolecular cross-links with another BBI. The heat-induced BBI inactivation mechanism was attributed to conformational change by β-elimination reactions (Cysteine residues of BBI were degraded which made disulfide bond breaking during the reaction) [23]. Furthermore, β-elimination reactions also can generate free SH and dehydroalanine which could create new intramolecular cross-links in BBI, changing the conformation of BBI [88].

#### 3.1.2. Microwave Processing

Microwave processing is a dielectric heating technique. In high-frequency electromagnetic fields, the temperature of material may rapidly increase due to the vibration, friction, and collision of polar molecules (primarily water) [89]. In comparison with conventional thermal techniques, microwaves can enhance inactivation rates of ANFs [90]. By establishing kinetic models, it was found that microwave technology had a higher inactivation efficiency for LOX and STIs than thermal processing [91]. It was reported that the reduction in STIs occurred after microwave treatment at 2450 MHz for 30 min [38]. The thermal effect of the microwave led to changes in the secondary structures and promoted the aggregation of STIs to inactive. The digestibility of soymilk rose to 93%, and the activity of STIs decreased to 17% following 10 min of microwave treatment at 85 °C [92]. Microwaves can generate non-thermal effects on food materials, e.g., electromagnetic fields and free radicals [93]. The oscillating electric fields can lead to the modification of protein secondary and tertiary structures [94]. The active center of STIs in soymilk could shift from fully hydrogen-bonded to partially hydrogen-bonded configuration under oscillating electric field, resulting in the alteration of hydrogen bond network configuration and the inactivation of STIs [95].

#### 3.1.3. Polyphenols

In recent years, studies have found that CIA can still be maintained about 89% by thermal treatments at 100 °C for 20 min, while polyphenols can effectively inactivate STIs during soymilk processing [87,96]. According to previous study, TIA and CIA were reduced from 788.3 ± 10.4 U/mL and 918.7 ± 18.0 U/mL to 388.3 ± 35.5 U/mL and 633.3 ± 52.8 U/mL, respectively, after adding 0.6 mg/g of tea polyphenols to soymilk [55]. Moreover, it was reported that EGCG and EGC in tea polyphenols also could reduce the activity of BBI, with inhibition rates of 51% and 21%, respectively [97]. Polyphenolic substances may change the inhibitory effect of BBI by forming the stable 1:1 complexes to inactivate CIA.

### 3.2. Maillard Reaction

Maillard reaction is a classic inactivation mechanism for STIs. The Maillard reaction involves the covalent binding of reducing sugars with amino groups of proteins (such as lysine and arginine), forming glycosylation products. This process significantly impacts the structure and function of STIs. It was reported that heating KTI with reducing sugars like glucose or lactose reduced the antigenicity of KTI by 60–80% at 120 °C, retaining 60% lysine [98]. It was indicated that lysine modification was the key mechanism driving the reduction in KTI antigenicity. The active center of KTI is composed of lysine residues. The activity of STIs is reduced by glycosylation modification masking or altering the spatial conformation of these key sites [99]. Additionally, the inactivation of BBI is associated with gradual oxidation modification of guanidine groups and reduction in free amino groups [20].

### 3.3. Novel Treatments

#### 3.3.1. Radio Frequency (RF)

Radio frequency (RF) refers to electromagnetic waves with frequencies ranging from 1 to 300 MHz. The sample is heated by converting electromagnetic energy into thermal energy through collisions with ions, molecules, and friction created by the heat [100]. It has faster heating rates and greater penetration capabilities compared with the conventional thermal methods [101]. Research [102] reported that RF treatment can generate better digestibility of proteins and lower activity of STIs (≥100 °C) at higher temperature, short time, and low frequency. The content of ANFs, e.g., STIs, tannins, saponins, and phytic acid in soybeans, could decrease after being treated with RF at 6 kW for 30 min [38]. The activities of STIs may be reduced significantly by about 95.2% after RF treatment at 27.12 MHz. The thermal effects of RF are responsible for the inactivation of STIs due to the changes in structure [103].

#### 3.3.2. Electric Fields

Applying a specific electric field to food can induce changes in the physicochemical properties of its electrically-sensitive constituents [104]. Researchers have proved that electric fields can inactivate STIs. The reason can be explained that the surface area of residues (especially tryptophan and cysteine residues) in STIs are largely exposed to the electric field. This makes STIs more able to interact with surrounding other molecules, resulting in the breaking of disulfide bonds and inactivation of the STIs [105]. Electric fields (Ohmic Heating (OH) and Pulsed Electric Fields (PEF)) are often used in inactivating STIs.

OH is a process in which an electric current passes through an electrical resistance to cause heat. Compared to traditional heating, it offers quick and uniform heating to reduce thermal damage to foods [106]. About 87% activity of STIs in soymilk could be inhibited at 50 Hz. The reason was that OH combines electrochemical and thermal effects. Protein disulfide bonds can be reduced by OH radicals through electrochemical reduction at lower temperatures. As the temperature increases, the thermal effect promotes inactivation of STIs [107,108]. Unlike OH, PEF induces irreversible modifications in proteins and enzymes by applying short high-voltage pulses to cause electroporation [92]. It could effectively reduce LOX and STIs (69.45% and 75.61%), respectively, at 40 kV/cm, 2250 ms in soymilk [109]. This is caused by the fact that PEF may cause the polarization of proteins, molecular unfolding, and change in molecular conformation, which promotes the inactivation of STIs.

#### 3.3.3. Infrared Heating (IRH)

Due to short time and high efficiency, IRH is extensively employed in food processing, e.g., drying, baking, blanching, pasteurization, sterilization, and the reduction in ANF. It can reduce the activity of urease and STIs in soybean proportional to IRF processing time and power [110]. Notably, the IRH combined with ultraviolet treatment could effectively inactivate LOX-1, LOX-3, and STIs, and reduce their activities by 55%, 97%, and 99%, respectively [111]. When IRH was integrated with soaking pretreatment, the inactivation efficiency of STIs could be increased to 95% [110]. Additionally, sprouting and IRH combination treatments could decrease the activity of STIs in soybean to 23%. These combined infrared technologies could also produce snacks like potato chips and ready-to-eat bean sprouts with lower levels of ANFs [111,112].

#### 3.3.4. Pressure Treatments

Pressure treatment affecting the activity of STIs has been discussed in current academic papers. The treatment can change the structures of STIs by affecting the covalent bonds. It also may change the physicochemical interactions between STI molecules in the system, including surface hydrophobicity and electrostatic interactions [113]. The most commonly used pressure treatments include High Pressure Processing (HPP), Ultra-high-pressure Homogenization (UHPH), and High Hydrostatic Pressure (HHP).

HPP can promote protein unfolding, retain nutrients and sensory qualities, and reduce TIA in soybean. [114]. It was reported that the LOX activity relative to beany and grassy flavors was decreased to 68.13 ± 12.80%, while TIA was only reduced to 91.30 ± 1.30% in soymilk after being treated by HPP at 600 MPa for 25 min [42]. Another study found that HPP slightly decreased the activity of STIs (about 10% or less). The mechanism of STIs inactivation by HPP is due to the collapse of the hydrophobic core, which induces molecular unfolding [115].

UHPH can significantly reduce the particle size of material and inactivate spoilage microorganisms and pathogens in food [116]. It produces shear forces, turbulence, and impact forces to affect allergens, ANFs, and other food components. During the processing of soymilk, the activity of soybean agglutinins can be reduced with pressure above 500 MPa [95]. And the activity of STIs could also be reduced to 37% with a pressure higher than 300 MPa [117]. This was attributed to the hydrodynamic cavitation (HC) produced by UHPH treatment. HC represented variations in liquid pressure and velocity due to bubble formation [118]. It could change the secondary structure and result in the inactivation of STIs.

HHP technology utilizes fluids as pressure transmission media to decrease the concentration of ANFs and allergens in soybean [119]. As reported by Linsberger-Martin, when HHP treatment was applied to soybean (600 MPa, 60 °C, 60 min), the activity of phytic acid, total phenolic acid, and STIs was reduced by 35.6%, 10.0%, and 98.5%, respectively [120]. The reason could be that STIs molecules penetrated by water molecules may have a more relaxed structure and expose additional reactive sites, which enhances their susceptibility to enzymatic hydrolysis and inactivation [121]. The SBA activity was reduced by 64% due to the destruction of its secondary and tertiary structures at 550 Pa for 15 min. Besides the inactivation efficiency of STIs, HHP is also a better preservation method of fresh food than thermal processing. However, the production cost of HHP is too high, which limits its wide application in the food industry.

### 3.4. Combinating Treatments

#### 3.4.1. Temperature–Pressure Treatment

High temperatures can cause loss of heat-sensitive nutrients in soybean, e.g., vitamins and soybean isoflavones [85]. Moreover, high temperature can also promote the coagulates and denatures of soybean protein, and the exudation of soybean-free fat [122]. Combining temperature with pressure treatments is effective in solving these problems. Temperature–pressure treatments including Thermal-high Hydrostatic Pressure (THHP) and Instant Controlled Pressure Drop treatment (DIC) are widely used in inactivation studies of STIs.

THHP is considered an environmentally friendly non-thermal technology that is used to improve the quality and freshness of food and reduce microbiological contamination [121]. THHP treatment could inactivate STIs and LOX in soymilk completely and enhance the physical stability and nutritional value of the product without chelating agents [123]. It was reported that THHP at 300 MPa and 75 °C produced commercially sterile soymilk with high physical stability compared with conventional thermal treatments [117]. In addition, researchers found that THHP at the temperatures of 77–90 °C and pressures of 525–750 MPa for less than 2 min can also achieve 90% inactivation of TIA. The reason for their inactivation is that the temperature–pressure disrupts the structure of aromatic rings, disulfide bonds, and β-sheets, leading to conformational changes and reduction in activity in STIs [43,114].

DIC is used for drying and expanding heat-sensitive crops and eliminating ANFs in biological materials [124]. It is a temperature–pressure technology that combines steam pressure (up to 8 bar) with temperature (up to 170 °C) for a short time (up to 3 min) [125]. Different STI reduction ratios could be achieved by DIC treatment depending on the operating parameters used, with higher pressures leading to lower STIs content. STIs activity in soybean (50% dry matter) can be decreased by 94% at 0.7 MPa for 1 min [44,126]. In another research, after DIC treatment (6 bar, 1 min), STIs were almost completely inactivated, and the ANFs, e.g., phytic acid and lectins, were also reduced [125,127].

#### 3.4.2. Extrusion Processing

Extrusion processing includes heat and mass transmission, mixing, shearing, particle size reduction, melting, texturizing, caramelizing, and shaping [128]. Different from traditional methods, extrusion processing is a complex thermo-mechanical process that requires precise conditions including the feed composition, moisture content, the cooking temperature along the extruder, and the die and screw speed [129]. Extrusion processing can improve the nutritional value of food by gelatinization of starch, reduction in lipid oxidation, enhancement of soluble dietary fiber, and reduction in ANFs [130]. Under specific extrusion conditions—18% raw material moisture content, 160 °C die temperature, and 200 rpm screw speed—a marked reduction in STIs, phytic acid, and tannins, about 99.54%, 99.30%, and 98.83%, respectively, could be observed [46]. However, extrusion processing may change the rheological and nutritional characteristics of the product. When extrusion processing is combined with different sequential processing methods (pre-cooking, pre-heating, humidification) to optimize the nutritional quality of the product. These processes almost completely inactivated STIs at lower temperatures, enhancing nutrient retention and improving sensory quality [129].

### 3.5. Other Treatments

#### 3.5.1. Ultrasonication

Ultrasonication processing can induce cavitation to inactivate STIs by generating high shear stress, releasing free radicals, and disrupting disulfide bonds [131]. BBI demonstrates strong ultrasound resistance due to stable disulfide bonds. Comparing with BBI, the disulfide bonds of KTI are easy to transform to terminal thiol group and change the secondary conformations of soybean protein [132].

The hydrogen and hydroxyl radicals generated by high-frequency ultrasound (200–800 kHz) can chemically damage proteins, which may lead to the loss of enzyme activity [133]. It was confirmed that ultrasound could rapidly oxidize methionine to reduce the activity of STIs at 355 kHz [134]. However, low-frequency ultrasound (e.g., 25 kHz) has strong physical forces (shear forces and microstreaming) that can change the conformation of enzymes by disrupting van der Waals’ interactions or hydrogen bonding [133]. Some researchers have also proven that the activity of STIs can be reduced to 48% at 25 kHz, 400 W, and 16 min [134]. The effects of single ultrasound treatment on the activity of STIs are limited. The combination treatment of ultrasound (60 °C, 30 min) with microwave (3 min) can decrease about 95% of TIA [135].

#### 3.5.2. Irradiation

Food irradiation is a typical non-thermal technique that involves exposing food or raw materials to high-energy and highly penetrating radiation, which causes chemical or physical modifications of food components [136]. Gamma irradiation and Electron beam irradiation (EBI) are the most commonly used irradiation techniques.

Gamma irradiation is known for its capacity to maintain freshness, eliminate pathogens, and diminish allergenic properties. It can induce changes in biomolecules conformational by causing oxidation, covalent bond cleavage, and the formation of free radicals. As the gamma irradiation dose increased to 60 kGy, the urease, LOX, and STIs could be completely inactivated [137]. EBI is a pollution-free new technology of irradiation. It can induce atoms or molecules in the material to collide with the electron beam by using a high-energy electron beam to produce free radicals, break chemical bonds, and exhibit other effects. This process can effectively remove bacteria, molds, yeasts, parasites, and ANFs to ensure the safety and shelf life of foods [103]. It was reported that phytic acid was reduced by 90% and the activity of trypsin inhibitors in canola meal was also decreased by EBI at 15 kGy [138]. Ebrahimi-Mahmoudabad [139] also reported that EBI could eliminate phytic acid and reduce 73% of STIs’ activity at 30 kGy. However, the FDA regulated that the irradiation was beneficial at 10 kGy or less on food [45]. The application of gamma irradiation is limited due to the safety and environment. It is necessary to connect other food processing technologies, such as heat treatment, and high-pressure processing to achieve greater inactivation efficiency.

#### 3.5.3. Dielectric-Barrier Discharge (DBD) Plasma

DBD plasma is a low-temperature (30–60 °C) treatment. Ionization of gases can produce plasma (ultraviolet radiation, negative ions, positive ions, ozone, free electrons, reactive oxygen species, and reactive nitrogen species) in the electric field, and change the biochemical properties of protein [140]. More than 80% of SBA was reduced by DBD at 40 kV, 4 min, while 84% of the STIs were inactivated in soymilk by DBD at 33.8 kV, 5 min. The degradation products of SBA and STIs are safe for the human body [47]. The inactivation of SBA and STIs is attributed to the bombardment of protein molecules by active particles in the electric field, which disrupts chemical bonds on the protein surface. Additionally, the particle collisions may generate radicals, which can oxidize surface amino acids and disrupt the protein structure [141,142]. Similarly, SBA activity was significantly reduced by plasma treatment, as indicated by hemagglutination assay and ELISA analysis. The inactivation mechanism of SBA activity was found to involve the oxidation of NH/NH2 of peptide bonds and subsequent modification of amino acid side chains, cleavage of peptide bonds, and breakage of polypeptides, leading to the oxidation of amino acids and molecule fragmentation of SBA [143].

### 3.6. Chemical Treatments

Chemical treatments can modify the molecular weight, chemical composition, and molecular architecture of protein [144]. Therefore, the amino acids at the active sites of STIs, such as lys and cysteine, as well as the important chemical bonds like disulfides and hydrogen bonds, are selectively functionalized and may undergo disruption to various extents which may cause changes in STIs activity [54]. The primary chemical treatments of inactivation mainly include acid-base salts, polyphenols, and reducing agents.

#### 3.6.1. Acid, Alkali, Salt

Soaking soybeans with acid, alkaline, or salt solutions is helpful to decrease the activity of STIs. Studies have shown that LOX activity was eliminated by soaking in 0.3 M HC1 at either 23 °C or 40 °C for 8 h, and less than 50% STIs remained, and urease was inactivated to an acceptable level [49]. The activity of STIs could also be decreased when soybeans were immersed in sodium hydroxide, ammonium hydroxide, and sodium bicarbonate solutions. Strong acids and alkalis can cause denaturation and inactivation of STIs in soybeans by changing amino acid charges, disrupting non-covalent bonds, and changing pH sensitivity at the active site [22,51]. NaCl is the main flavoring in our daily life. Its solution can accelerate the KTI incorporation into protein aggregates and the cleavage of the BBI peptide bond. The process would provide a simple and quick method for the processing of low TIA soymilk [22]. Considering safety, soybeans are usually soaked with only a small amount of sodium bicarbonate, sodium chloride, or water in daily life.

#### 3.6.2. Reducing Agents

The underlying theory of reductant inactivation of STIs by reducing agents has been broadly categorized into two aspects: one is the cleavage of disulfide bonds, and the other is the destruction or modification of specific amino acid residues.

Some reducing agents can cleave disulfide bonds to affect the activity of STIs [145]. Different reducing agents have different inactivation efficiency. Sodium metabisulfite and L-cysteine have little effect on the reductions in TIA in soybean flour [52]. Alina Rehder reported that using zinc as a reducing agent can significantly reduce the inhibitory activity of KTI and BBI by 72% and 85% [53]. However, when thermal treatment was combined with reducing agents, the inactivation efficiency of STIs in soybean flour could reach 99%. This effect was attributed to the fact that higher temperature can promote the reduction or rearrangement of disulfide bonds in STIs, resulting in the complete unfolding of most STIs and decreasing their biological activity [50].

The activity of STIs is directly influenced by specific amino acids at the active site, including lysine, arginine, and serine. The specificity of STIs being targeted is often reflected by the amino acid residues within the active site of the inhibitor. A variety of reducing agents, e.g., dimethylacetamide and isopropanol have been shown that could change the charge and network of amino acid residues, thus affecting the structural and functional characteristics of STIs [16]. Lysine in STIs was acetylated, which made STIs inactivated [54]. This is due to the reaction between maleic anhydride and the amino groups of lysine, which results in peptide bond breakage within KTI. Comparable effects were also found in methanol treatment of STIs. The reasons were also attributed to the interaction between methanol and critical amino acid residues, e.g., arginine and isoleucine at the active site [16]. This interaction changed the microenvironment of these residues, reducing the inhibitor’s capacity to bind with the enzyme. However, the application of some reducing agents could represent and health concern and, thus, would affect food safety. We recommend combining them with thermal/non-thermal treatments and prioritizing food-grade agents like sodium metabisulfite.

### 3.7. Biological Treatments

Biological methods, e.g., enzymatic degradation, germination, and fermentation, are effective strategies for inactivating STIs in soybeans. These technologies can reduce the activity of STIs through biochemical reactions and microbial metabolism. Comparing with thermal treatments, biological methods can preserve the sulfur-containing amino acids that are primarily found in various types of protease inhibitors in legume proteins [146]. Enzymolysis has gentle conditions, which can avoid the destruction or racemization of amino acid side chains and improve amino acid bioavailability [147]. Alkaline protease can reduce the blood-clotting activity and antinutritional effects of SBA by hydrolyzing its quaternary structure while generating bioactive peptides with antihypertensive and antioxidant properties [148]. During the germination of soybeans, the activity of STIs can also be decreased by activating endogenous proteases. STIs can be degraded to amino acids for early seed growth [149]. Several researchers also found that a variety of bacteria, e.g., *Lactobacillus acidophilus*, *Bacillus subtilis*, and *Lactobacillus bulgaricus*, can also produce enzymes to reduce the activity of STIs [150,151,152]. The fermentation of the combination of multiple probiotics and prebiotics may be more efficient in reducing STIs activity, which needs further study.

## 4. Conclusions and Future Perspectives

This review provides an in-depth analysis of the structural properties of KTI and BBI and their inhibitory effects on trypsin activity during the digestive process. By comparing the parameters and effects of different treatment techniques, it was found that physical (e.g., heating, ultrasound, and high pressure) and chemical (e.g., acid, alkaline, salts, and reducing agents) treatments were effective in reducing the activity of STIs. However, they have harmful effects on the sensory quality and nutrient content of the food. Because of the limitations of single technology, this paper emphasizes the importance of combining multiple technologies, which can not only improve the inactivation efficiency of STIs, but also better maintain the quality of food products. In addition, this paper also explores the molecular mechanisms underlying the reduced activity of STIs, especially the structural changes induced by disulfide bond breakage and protein aggregation, which are important for understanding the STIs inactivation process.

**Figure 5:**
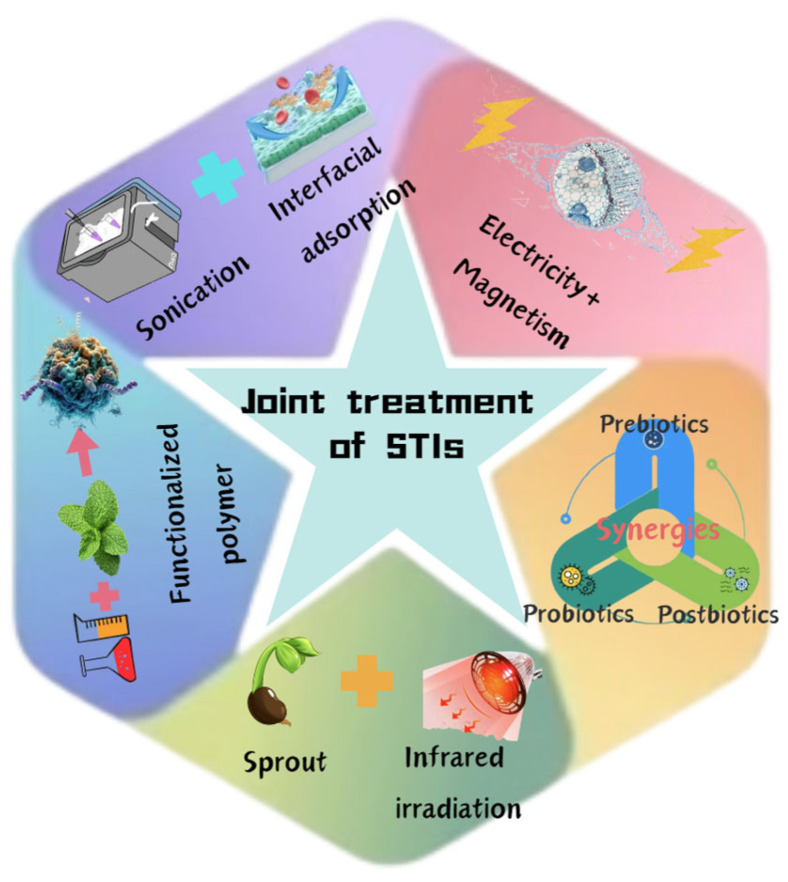
Future joint treatment processing of inactivating Soybean Trypsin Inhibitors (STIs).

More methods will be discovered for STIs inactivation in the future. For thermal processing, researchers can use immunofluorescence to observe the ultrastructural distribution of STIs and soybean proteins. They can also use immunoprecipitation and crosslinking mass spectrometry to study the proteomics of STIs and soybean protein fractions interactions (7 s, 11 s). At the molecular level, molecular docking, computational modeling, and spectroscopic analysis will be used to analyze the active site changes of TIA and CIA and explore the dynamics of amino acids and disulfide bonds. The safety assessment of products is worth exploring. In vitro digestion experiments, mouse experiments, and epidemiological investigations are trusted validation methods. Additionally, research will be focused on developing combined processing techniques, including the synthesis of specific functional polymers, combination of thermal and non-thermal treatments, and complex action of multiple proteases to improve STIs inactivation efficiency (Figure 5). Balancing the reduction in substances that are not good for nutrition and increasing the nutrients that are good for health is a difficult task. This will be a key point of research.

## Figures and Tables

**Table 1:** Physical treatments.

Treatment	Conditions	Advantage	Disadvantage	Studies
Cooking on the stove	20 min	Improves protein digestibility in vitro	Destroy other nutrients	[36]
Microwave	2450 MHZ, 4 min	Short term and lower energy	Lower inactivation efficiency than cooking	[37,38]
Pressure and cooking	120 °C, 10 min	——	Retaining more BBI	[37]
Regular hot air drying	100 °C, 2 h	——	——	[39]
Fluidized bed drying	Initial inlet air temperature of 80 °C, 30 min after processing temperature 60 °C instead to continue processing	Ensuring stable product properties	Higher energy consumption	[40]
Ohmic heating	220 V, 50 Hz	Reducing allergenicity Ensuring protein quality	——	[41]
High-pressure processing	600 MPa, 25 min	Reducing the content of ANFs	Low inactivation efficiency for STIs	[42]
High pressure and heating	Various combinations of temperature (300 K, 345 K, and 373 K) and pressure (1 bar, 3 kbar, and 6 kbar)	Maintaining quality and freshness of foods High inactivation efficiency	——	[43]
Instant Controlled Pressure Drop	1 min, 6 min	Highly effective in inactivating ANFs	Lossing of heat-sensitive nutrients	[44]
Ultrasonic treatment	25 kHz, 400 W, 16 min	Maintaining the nutrients and flavor of food	Less impact on BBI	[43]
Irradiation	10 kGy or less irradiation	Short-term and highly efficient	Causing security problems	[45]
Extrusion processing	160 °C of mold temperature and screw speed of 200 rpm	Achieving complete inactivation of ANFs	Changing the rheological and nutritional characteristics	[46]
Dielectric-barrier discharge plasma	23 V, 15 min or 33.8 KV, 5 min	Improving protein solubility and emulsification Reducing STIs by more than 80%	——	[47,48]

Note: “——” indicates not recorded or no data.

**Table 2:** Chemical treatments.

Treatment	Conditions	Advantage	Disadvantage	Studies
Acids, alkalis, and salt	HCl, 23 °C or 40 °C, 8 h	Rapiding reaction High efficiency	Nutritional Loss. Deterioration of sensory quality. Environmental Concerns.	[49]
	Nacl, heating	Simplicity and ease of using. Low Cost.	Limiting inactivation effect, Impacting on sensory quality.	[21]
	NaoH	Rapiding reaction High efficiency	Nutritional Loss. Deterioration of sensory quality. Environmental Concerns.	[50]
	NaHCO_3_	Mild Processing Conditions	Incompleting inactivation	[51]
Disulfide bond modification	Sodium metabisulfite or L-cysteine, 25 °C, 2 h	Combining treatment completely inactivates SITs	——	[52]
	Zn	Suitable for industrial inactivation	Heavy metal poisoning	[53]
Modification of amino acid residues	Maleicanhydride, pH = 3.5–9.5, 30 min	——	Chemical residues are harmful to health.	[54]
	CH_3_OH	——	Chemical residues are harmful to health	[16]
Polyphenols	TPs	Improving nutritional value and antioxidant properties	Influence on product flavor	[55]
	Stevioside	Effective inactivation of BBI Increasing product flavor	Expensive	[24]

Note: “——” indicates not recorded or no data.

**Table 3:** Biological treatments.

Treatment	Conditions	Advantage	Disadvantage	Studies
Enzymolysis	Alcalase, pH = 8, 60 °C, 4 h	Specificity and mild reaction conditions	Producing by-products	[56]
High-pressure homogenization-assisted enzymatic digestion	——	Process controllable Improving food texture and stability	Affecting by multiple conditions. The process is complex	[57]
Germination	3 d 32 °C, 90% relative humidity	Simple and easy, does not require equipment Green and safe Increasing the content of soluble sugar, vitamins, and trace elements	Longer time and difficult to store The nutrient composition changes	[58]
Fermenting	Acidophilus, Bacillus subtilis, Lactobacillus bulgaricus, 5 d	Improving the bioavailability of proteins and peptides	——	[59]

Note: “——” indicates not recorded or no data.

## Data Availability

No new data were created or analyzed in this study. Data sharing is not applicable to this article.

## Supplementary Materials

The following supporting information can be downloaded at: https://www.mdpi.com/article/10.3390/foods14060975/s1, Table S1. Various antinutritional factors and their harmful effects on the human body.

## References

[1] Padalkar G., Mandlik R., Sudhakaran S., Vats S., Kumawat S., Kumar V., Kumar V., Rani A., Ratnaparkhe M.B., Jadhav P. (2023). Necessity and challenges for exploration of nutritional potential of staple-food grade soybean. J. Food Compos. Anal..

[2] Anggraeni A.A., Triwitono P., Lestari L.A., Harmayani E. (2024). Evaluation of glucomannan as a fat replacer in the dough and cookies made from fermented cassava flour and soy protein concentrate. Food Chem..

[3] Liu X., Sala G., Scholten E. (2024). Impact of soy protein dispersibility on the structural and sensory properties of fat-free ice cream. Food Hydrocoll..

[4] Zeng X., Li Y., Li P., Zhao J., Li X., Wang X., Liu B., Ni L., Li H., Xi Y. (2024). Encapsulation of roast beef flavor by soy protein isolate/chitosan complex pickering emulsions to improve its releasing properties during the processing of plant-based meat analogues. Food Chem..

[5] Singh P., Kumar R., Sabapathy S., Bawa A. (2008). Functional and edible uses of soy protein products. Compr. Rev. Food Sci. Food Saf..

[6] Gong H., Fu H., Zhang J., Zhang Q., Wang Y., Wang D., Cai L., Chen J., Yu H., Lyu B. (2024). Preparation of soybean protein-based nanoparticles and its application as encapsulation carriers of bioactive substances. LWT.

[7] Chen J., Mu T., Goffin D., Blecker C., Richard G., Richel A., Haubruge E. (2019). Application of soy protein isolate and hydrocolloids based mixtures as promising food material in 3d food printing. J. Food Eng..

[8] Zhang J., Long Y., Zhang Y., Lu L., Liu C., Xu W., Hu Z., Hu C. (2025). Study on the effect of heat treatment process on the quality characteristics of soybean-citri reticulatae pericarpium (crp) based yogurt and related mechanisms. Food Hydrocoll..

[9] Fu J., Zhan Z., Duan Q., Yang Y., Xie H., Dong X., Zhang H., Yu L. (2025). Application of soybean protein isolates-polysaccharides hybrid emulsion gels as alternative fats in fabricating plant-based meats with two-phase. LWT.

[10] Tkaczewska J., Jamróz E., Zając M., Guzik P., Gedif H.D., Turek K., Kopeć M. (2023). Antioxidant edible double-layered film based on waste from soybean production as a vegan active packaging for perishable food products. Food Chem..

[11] Liu K. (2012). Soybeans: Chemistry, Technology, and Utilization.

[12] Hu M., Gao Y., Wen W., Zhang P., Zhang F., Fan B., Wang F., Li S. (2024). The aggregation behavior between soybean whey protein and polysaccharides of diverse structures and their implications in soybean isoflavone delivery. Food Chem..

[13] Francis G., Makkar H.P., Becker K. (2001). Antinutritional factors present in plant-derived alternate fish feed ingredients and their effects in fish. Aquaculture.

[14] Li S., Sauer W.C., Huang S., Hardin R.T. (1998). Response of pancreatic secretions to feeding diets with low and high levels of soybean trypsin inhibitors in growing pigs. J. Sci. Food Agric..

[15] Häußler D., Scheidt T., Stirnberg M., Steinmetzer T., Gütschow M. (2015). A bisbenzamidine phosphonate as a janus-faced inhibitor for trypsin-like serine proteases. ChemMedChem.

[16] Li C., Li W., Zhang Y., Simpson B.K. (2020). Comparison of physicochemical properties of recombinant buckwheat trypsin inhibitor (rbti) and soybean trypsin inhibitor (sbti). Protein Expr. Purif..

[17] Vagadia B.H., Vanga S.K., Raghavan V. (2017). Inactivation methods of soybean trypsin inhibitor—A review. Trends Food Sci. Technol..

[18] Song H., Suh S. (1998). Kunitz-type soybean trypsin inhibitor revisited: Refined structure of its complex with porcine trypsin reveals an insight into the interaction between a homologous inhibitor from Erythrina caffra and tissue-type plasminogen activator. J. Mol. Biol..

[19] Koide T., Ikenaka T. (2010). Studies on Soybean Trypsin Inhibitors. 3. Amino-Acid Sequence of the Carboxyl-Terminal Region and the Complete Amino-Acid Sequence of Soybean Trypsin Inhibitor (Kunitz). Eur. J. Biochem..

[20] Losso J.N. (2008). The biochemical and functional food properties of the bowman-birk inhibitor. Crit. Rev. Food Sci. Nutr..

[21] Birk Y. (1985). The Bowman-Birk inhibitor. Trypsin-and chymotrypsin-inhibitor from soybeans. Int. J. Pept. Protein Res..

[22] Chen Y., Xu Z., Zhang C., Kong X., Hua Y. (2014). Heat-induced inactivation mechanisms of Kunitz trypsin inhibitor and Bowman-Birk inhibitor in soymilk processing. Food Chem..

[23] He H., Li X., Kong X., Hua Y., Chen Y. (2017). Heat-induced inactivation mechanism of soybean Bowman-Birk inhibitors. Food Chem..

[24] Liu C., Luo L., Wu Y., Yang X., Dong J., Luo F., Zou Y., Shen Y., Lin Q. (2019). Inactivation of soybean Bowman–Birk inhibitor by stevioside: Interaction studies and application to soymilk. J. Agric. Food Chem..

[25] Nieto-Veloza A., Wang Z., Zhong Q., D’Souza D., Krishnan H.B., Dia V.P. (2022). Lunasin protease inhibitor concentrate decreases pro-inflammatory cytokines and improves histopathological markers in dextran sodium sulfate-induced ulcerative colitis. Food Sci. Hum. Wellness.

[26] Li Q., Huang L., Luo Z., Tamer T.M. (2020). Stability of trypsin inhibitor isolated from potato fruit juice against ph and heating treatment and in vitro gastrointestinal digestion. Food Chem..

[27] Cruz-Huerta E., Fernández-Tomé S., Arques M.C., Amigo L., Recio I., Clemente A., Hernández-Ledesma B. (2015). The protective role of the bowman-birk protease inhibitor in soybean lunasin digestion: The effect of released peptides on colon cancer growth. Food Funct..

[28] Fang E.F., Wong J.H., Ng T.B. (2010). Thermostable kunitz trypsin inhibitor with cytokine inducing, antitumor and hiv-1 reverse transcriptase inhibitory activities from korean large black soybeans. J. Biosci. Bioeng..

[29] de Siqueira Patriota L.L., Ramos D.d.B.M., e Silva M.G., dos Santos A.C.L.A., Silva Y.A., de Oliveira Marinho A., Coelho L.C.B.B., Paiva P.M.G., Pontual E.V., Mendes R.L. (2021). The trypsin inhibitor from moringa oleifera flowers (mofti) inhibits acute inflammation in mice by reducing cytokine and nitric oxide levels. S. Afr. J. Bot..

[30] Kennedy A.R. (1998). The bowman-birk inhibitor from soybeans as an anticarcinogenic agent. Am. J. Clin. Nutr..

[31] Kuenz S., Thurner S., Hoffmann D., Kraft K., Wiltafsky-Martin M., Damme K., Windisch W., Brugger D. (2022). Effects of gradual differences in trypsin inhibitor activity on the estimation of digestible amino acids in soybean expellers for broiler chickens. Poult. Sci..

[32] Aderibigbe A., Cowieson A., Ajuwon K., Adeola O. (2021). Contribution of purified soybean trypsin inhibitor and exogenous protease to endogenous amino acid losses and mineral digestibility. Poult. Sci..

[33] Embaby H.E.-S. (2010). Effect of heat treatments on certain antinutrients and in vitro protein digestibility of peanut and sesame seeds. Food Sci. Technol. Res..

[34] Cabrera-Orozco A., Jiménez-Martínez C., Dávila-Ortiz G. (2013). Soybean: Non-nutritional factors and their biological functionality. Soybean-Bio-Act. Compd..

[35] Fasina Y., Garlich J., Classen H., Ferket P., Havenstein G., Grimes J., Qureshi M., Christensent V. (2004). Response of turkey poults to soybean lectin levels typically encountered in commercial diets. 1. Effect on growth and nutrient digestibility. Poult. Sci..

[36] Zhang Y., Chang S.K. (2022). Trypsin inhibitor activity, phenolic content and antioxidant capacity of soymilk as affected by grinding temperatures, heating methods and soybean varieties. LWT.

[37] Andrade J., Mandarino J., Kurozawa L., Ida E. (2016). The effect of thermal treatment of whole soybean flour on the conversion of isoflavones and inactivation of trypsin inhibitors. Food Chem..

[38] Zhong Y., Wang Z., Zhao Y. (2015). Impact of radio frequency, microwaving, and high hydrostatic pressure at elevated temperature on the nutritional and antinutritional components in black soybeans. J. Food Sci..

[39] Agrahar-Murugkar D., Jha K. (2010). Effect of drying on nutritional and functional quality and electrophoretic pattern of soyflour from sprouted soybean (*Glycine max*). J. Food Sci. Technol..

[40] Cheng S., Langrish T.A. (2023). Fluidized bed drying of chickpeas: Developing a new drying schedule to reduce protein denaturation and remove trypsin inhibitors. J. Food Eng..

[41] Wang L., Li D., Tatsumi E., Liu Z., Chen X., Li L. (2007). Application of two-stage ohmic heating to tofu processing. Chem. Eng. Process. Process. Intensif..

[42] Rajan A., Velusamy M., Baskaran K., Rangarajan J., Natarajan V., Radhakrishnan M. (2023). High pressure processing of whole soymilk: Effect on allergenicity, anti-nutritional factor, lipoxygenase activity and E-nose-aroma characteristics. Food Chem. Adv..

[43] Vanga S.K., Singh A., Raghavan V. (2018). Changes in soybean trypsin inhibitor by varying pressure and temperature of processing: A molecular modeling study. Innov. Food Sci. Emerg. Technol..

[44] Haddad J., Allaf K. (2007). A study of the impact of instantaneous controlled pressure drop on the trypsin inhibitors of soybean. J. Food Eng..

[45] Tewari K., Kumari S., Vinutha T., Singh B., Dahuja A. (2015). Gamma irradiation induces reduction in the off-flavour generation in soybean through enhancement of its antioxidant potential. J. Radioanal. Nucl. Chem..

[46] Rathod R.P., Annapure U.S. (2016). Effect of extrusion process on antinutritional factors and protein and starch digestibility of lentil splits. LWT—Food Sci. Technol..

[47] Xu Y., Sun Y., Huang K., Li J., Zhong C., He X. (2022). Inactivation of soybean trypsin inhibitor by dielectric-barrier discharge plasma and its safety evaluation and application. Foods.

[48] Dabade A., Kahar S., Acharjee A., Bhushette P., Annapure U. (2023). Effect of atmospheric pressure non-thermal pin to plate cold plasma on structural and functional properties of soy protein isolate. J. Agric. Food Res..

[49] Che Man Y., Wei L., Nelson A., Yamashita N. (1991). Effects of soaking soybeans in dilute acids on biologically active components. J. Am. Oil Chem. Soc..

[50] Wedemeyer W.J., Welker E., Narayan M., Scheraga H.A. (2000). Disulfide bonds and protein folding. Biochemistry.

[51] Baker E., Mustakas G. (1973). Heat inactivation of trypsin inhibitor, lipoxygenase and urease in soybeans: Effect of acid and base additives. J. Am. Oil Chem. Soc..

[52] Avilés-Gaxiola S., Chuck-Hernández C., del Refugio Rocha-Pizaña M., García-Lara S., López-Castillo L.M., Serna-Saldívar S.O. (2018). Effect of thermal processing and reducing agents on trypsin inhibitor activity and functional properties of soybean and chickpea protein concentrates. LWT.

[53] Rehder A., Sørensen J.C., Markedal K.E., Sørensen H., Sørensen S., Petersen I.L. (2021). Targeted inactivation of soybean proteinase inhibitors using zinc. Food Chem..

[54] Kong X., Li Y., Liu X. (2023). Purification of soybean Kunitz trypsin inhibitor and the mechanism of its passivation by lysine and disulfide bond modifications. Food Biosci..

[55] Ge G., Guo W., Zheng J., Zhao M., Sun W. (2021). Effect of interaction between tea polyphenols with soymilk protein on inactivation of soybean trypsin inhibitor. Food Hydrocoll..

[56] Rackis J.J. (1974). Biological and physiological factors in soybeans. J. Am. Oil Chem. Soc..

[57] Johnson R.A., Jakobs K.H., Schultz G. (1985). Extraction of adenylate cyclase-activating factor of bovine sperm and its identification as trypsin-like protease. J. Biol. Chem..

[58] Sharma S., Sahni P. (2021). Dynamics of germination behaviour, protein secondary structure, technofunctional properties, antinutrients, antioxidant capacity and mineral elements in germinated dhaincha. Food Technol. Biotechnol..

[59] Adeyemo S.M., Onilude A.A. (2013). Enzymatic reduction of anti-nutritional factors in fermenting soybeans by *Lactobacillus plantarum* isolates from fermenting cereals. Niger. Food J..

[60] Salim R., Nehvi I.B., Mir R.A., Tyagi A., Ali S., Bhat O.M. (2023). A review on anti-nutritional factors: Unraveling the natural gateways to human health. Front. Nutr..

[61] Herreman L., Nommensen P., Pennings B., Laus M.C. (2020). Comprehensive overview of the quality of plant-And animal-sourced proteins based on the digestible indispensable amino acid score. Food Sci. Nutr..

[62] Orborne T.B. (1924). The Vegetable Proteins. Nature.

[63] Tandang-Silvas M.R.G., Tecson-Mendoza E.M., Mikami B., Utsumi S., Maruyama N. (2011). Molecular design of seed storage proteins for enhanced food physicochemical properties. Annu. Rev. Food Sci. Technol..

[64] Maruyama N., Mikami B., Utsumi S. (2011). The development of transgenic crops to improve human health by advanced utilization of seed storage proteins. Biosci. Biotechnol. Biochem..

[65] Mori T., Maruyama N., Nishizawa K., Higasa T., Yagasaki K., Ishimoto M., Utsumi S. (2004). The composition of newly synthesized proteins in the endoplasmic reticulum determines the transport pathways of soybean seed storage proteins. Plant J..

[66] Sui X., Zhang T., Jiang L. (2021). Soy protein: Molecular structure revisited and recent advances in processing technologies. Annu. Rev. Food Sci. Technol..

[67] Taliercio E., Loveless T., Turano M.J. (2015). Identification of epitopes of the A1aBx and A5A4B3 subunits of glycinin antigenic in three animal species. Food Agric. Immunol..

[68] Adachi M., Takenaka Y., Gidamis A.B., Mikami B., Utsumi S. (2001). Crystal structure of soybean proglycinin A1aB1b homotrimer. J. Mol. Biol..

[69] Hughes G.J., Ryan D.J., Mukherjea R., Schasteen C.S. (2011). Protein digestibility-corrected amino acid scores (PDCAAS) for soy protein isolates and concentrate: Criteria for evaluation. J. Agric. Food Chem..

[70] Ashaolu T.J., Greff B., Varga L. (2024). The structure–function relationships and techno-functions of β-conglycinin. Food Chem..

[71] Zahir M., Fogliano V., Capuano E. (2018). Food matrix and processing modulate in vitro protein digestibility in soybeans. Food Funct..

[72] Han K., Feng G., Li T., Deng Z., Zhang Z., Wang J., Yang X. (2022). Digestion Resistance of Soybean 7S Protein and Its Implications for Reinforcing the Gastric Mucus Barrier. J. Agric. Food Chem..

[73] Rio A.R.D., Boom R.M., Janssen A.E.M. (2022). Effect of Fractionation and Processing Conditions on the Digestibility of Plant Proteins as Food Ingredients. Foods.

[74] Lin L., Jiao M., Zhao M., Sun W. (2019). In vitro gastrointestinal digest of catechin-modified β-conglycinin oxidized by lipoxygenase-catalyzed linoleic acid peroxidation. Food Chem..

[75] De Muelenaere H. (1964). Studies on the digestion of soybeans. J. Nutr..

[76] Krogdahl A., Holm H. (1981). Soybean proteinase inhibitors and human proteolytic enzymes: Selective inactivation of inhibitors by treatment with human gastric juice. J. Nutr..

[77] Laskowski M., Kato I. (1980). Protein inhibitors of proteinases. Annu. Rev. Biochem..

[78] Huber R., Kukla D., Bode W., Schwager P., Bartels K., Deisenhofer J., Steigemann W. (1974). Structure of the complex formed by bovine trypsin and bovine pancreatic trypsin inhibitor: II. Crystallographic refinement at 1.9 Å resolution. J. Mol. Biol..

[79] Polticelli F., Ascenzi P., Bolognesi M., Honig B. (1999). Structural determinants of trypsin affinity and specificity for cationic inhibitors. Protein Sci..

[80] Janin J., Chothia C. (1976). Stability and specificity of protein-protein interactions: The case of the trypsin-trypsin inhibitor complexes. J. Mol. Biol..

[81] Vincent J.P., Lazdunski M. (1972). Trypsin-pancreatic trypsin inhibitor association. Dynamics of the interaction and role of disulfide bridges. Biochemistry.

[82] Fersht A.R. (1974). Catalysis, binding and enzyme-substrate complementarity. Proc. R. Soc. Lond. Ser. B Biol. Sci..

[83] Tsunogae Y., Tanaka I., Yamane T., Kikkawa J., Ashida T., Ishikawa C., Watanabe K., Nakamura S., Takahashi K. (1986). Structure of the trypsin-binding domain of Bowman-Birk type protease inhibitor and its interaction with trypsin. J. Biochem..

[84] Tang C. (2021). Effect of Temperature on Molecular Conformation and Immunoreactivity of Soybean Lectin. Ph.D. Thesis.

[85] Machado F., Queiróz J., Oliveira M., Piovesan N., Peluzio M., Costa N., Moreira M. (2008). Effects of heating on protein quality of soybean flour devoid of kunitz inhibitor and lectin. Food Chem..

[86] Finley J.W., Wheeler E.L., Witt S.C. (1981). Oxidation of glutathione by hydrogen peroxide and other oxidizing agents. J. Agric. Food Chem..

[87] Xu Z., Chen Y., Zhang C., Kong X., Hua Y. (2012). The heat-induced protein aggregate correlated with trypsin inhibitor inactivation in soymilk processing. J. Agric. Food Chem..

[88] Volkin D.B., Klibanov A.M. (1987). Thermal destruction processes in proteins involving cystine residues. J. Biol. Chem..

[89] Guzik P., Kulawik P., Zając M., Migdał W. (2022). Microwave applications in the food industry: An overview of recent developments. Crit. Rev. Food Sci. Nutr..

[90] Kala B., Mohan V. (2012). Effect of microwave treatment on the antinutritional factors of two accessions of velvet bean, *Mucuna pruriens* (L.) DC. var. utilis (Wall. ex Wight) Bak. ex Burck. Int. Food Res. J..

[91] Kubo M.T., dos Reis B.H., Sato L.N., Gut J.A. (2021). Microwave and conventional thermal processing of soymilk: Inactivation kinetics of lipoxygenase and trypsin inhibitors activity. LWT.

[92] Vanga S.K., Wang J., Jayaram S., Raghavan V. (2021). Effects of pulsed electric fields and ultrasound processing on proteins and enzymes: A review. Processes.

[93] Kubo M.T., Siguemoto É.S., Funcia E.S., Augusto P.E., Curet S., Boillereaux L., Sastry S.K., Gut J.A. (2020). Non-thermal effects of microwave and ohmic processing on microbial and enzyme inactivation: A critical review. Curr. Opin. Food Sci..

[94] El Mecherfi K.E., Curet S., Lupi R., Larré C., Rouaud O., Choiset Y., Rabesona H., Haertlé T. (2019). Combined microwave processing and enzymatic proteolysis of bovine whey proteins: The impact on bovine β-lactoglobulin allergenicity. J. Food Sci. Technol..

[95] Han X., Sun Y., Huangfu B., He X., Huang K. (2023). Ultra-high-pressure passivation of soybean agglutinin and safety evaluations. Food Chem. X.

[96] Yuan S., Chang S.K., Liu Z., Xu B. (2008). Elimination of trypsin inhibitor activity and beany flavor in soy milk by consecutive blanching and ultrahigh-temperature (UHT) processing. J. Agric. Food Chem..

[97] Chen Z., Chen Y., Xue Z., Gao X., Jia Y., Wang Y., Lu Y., Zhang J., Zhang M., Chen H. (2020). Insight into the inactivation mechanism of soybean Bowman-Birk trypsin inhibitor (BBTI) induced by epigallocatechin gallate and epigallocatechin: Fluorescence, thermodynamics and docking studies. Food Chem..

[98] Oste R.E., Brandon D.L., Bates A.H., Friedman M. (1990). Effect of maillard browning reactions of the kunitz soybean trypsin inhibitor on its interaction with monoclonal antibodies. J. Agric. Food Chem..

[99] Hodge J.E. (1953). Dehydrated foods, chemistry of browning reactions in model systems. J. Agric. Food Chem..

[100] Mao Y., Wang S. (2021). Simultaneous hot-air assisted radio frequency drying and disinfestation for in-shell walnuts using a two-stage strategy. LWT.

[101] Jiao S., Johnson J., Tang J., Wang S. (2012). Industrial-scale radio frequency treatments for insect control in lentils. J. Stored Prod. Res..

[102] Takács K., Szabó E.E., Nagy A., Cserhalmi Z., Falusi J., Gelencsér É. (2022). The effect of radiofrequency heat treatment on trypsin inhibitor activity and in vitro digestibility of soybean varieties (*Glycine max*.(L.) Merr.). J. Food Sci. Technol..

[103] Ye X., Jiang S., Niu W., Bai R., Yang C., Wang S., Li Z., Zhang L., Han H., Xi J. (2024). Glycosylated gelatin prepared based on electron beam irradiation and its physicochemical properties. Int. J. Biol. Macromol..

[104] Niu D., Zeng X., Ren E., Xu F., Li J., Wang M., Wang R. (2020). Review of the application of pulsed electric fields (PEF) technology for food processing in China. Food Res. Int..

[105] Vagadia B.H., Vanga S.K., Singh A., Raghavan V. (2016). Effects of thermal and electric fields on soybean trypsin inhibitor protein: A molecular modelling study. Innov. Food Sci. Emerg. Technol..

[106] Guo W., Llave Y., Jin Y., Fukuoka M., Sakai N. (2017). Mathematical modeling of ohmic heating of two-component foods with non-uniform electric properties at high frequencies. Innov. Food Sci. Emerg. Technol..

[107] Dos Santos I.F., Pimentel T.C., da Cruz A.G., Stringheta P.C., Martins E., Campelo P.H. (2024). Ohmic Heating in Food Processing: An Overview of Plant-Based Protein Modification. Processes.

[108] Lu L., Zhao L., Zhang C., Kong X., Hua Y., Chen Y. (2015). Comparative effects of ohmic, induction cooker, and electric stove heating on soymilk trypsin inhibitor inactivation. J. Food Sci..

[109] Anbarasan R., Jaganmohan R., Anandakumar S., Mahendran R. (2023). Pulsed electric field treatment of soymilk: Impact on Kunitz trypsin inhibitor allergenicity, antinutritional factor, and aroma characteristics. J. Food Sci..

[110] Yalcin S., Basman A. (2015). Effects of infrared treatment on urease, trypsin inhibitor and lipoxygenase activities of soybean samples. Food Chem..

[111] Maetens E., Hettiarachchy N., Dewettinck K., Horax R., Moens K. (2018). Reductions of anti-nutritional factors of germinated soybeans by ultraviolet and infrared treatments for snack chips preparation. LWT.

[112] Sakare P., Giri S.K., Kate A. (2023). Optimization of sprouting and infrared radiation combination treatment for production of ready-to-eat sprouted soybean: Ready-to-eat sprouted soybean process optimization. J. Sci. Ind. Res. (JSIR).

[113] Avelar Z., Vicente A.A., Saraiva J.A., Rodrigues R.M.M. (2021). The role of emergent processing technologies in tailoring plant protein functionality: New insights. Trends Food Sci. Technol..

[114] Van Der Ven C., Matser A.M., Van den Berg R.W. (2005). Inactivation of soybean trypsin inhibitors and lipoxygenase by high-pressure processing. J. Agric. Food Chem..

[115] Hall A.E., Moraru C.I. (2021). Effect of High Pressure Processing and heat treatment on in vitro digestibility and trypsin inhibitor activity in lentil and faba bean protein concentrates. LWT.

[116] Masson L.M., Rosenthal A., Calado V.M., Deliza R., Tashima L. (2011). Effect of ultra-high pressure homogenization on viscosity and shear stress of fermented dairy beverage. LWT—Food Sci. Technol..

[117] Poliseli-Scopel F.H., Hernández-Herrero M., Guamis B., Ferragut V. (2012). Comparison of ultra high pressure homogenization and conventional thermal treatments on the microbiological, physical and chemical quality of soymilk. LWT—Food Sci. Technol..

[118] Suslick K.S., Mdleleni M.M., Ries J.T. (1997). Chemistry induced by hydrodynamic cavitation. J. Am. Chem. Soc..

[119] Zhang H., Feng X., Liu S., Ren F., Wang J. (2023). Effects of high hydrostatic pressure on nutritional composition and cooking quality of whole grains and legumes. Innov. Food Sci. Emerg. Technol..

[120] Linsberger-Martin G., Weiglhofer K., Phuong T.P.T., Berghofer E. (2013). High hydrostatic pressure influences antinutritional factors and in vitro protein digestibility of split peas and whole white beans. LWT—Food Sci. Technol..

[121] Garcia-Mora P., Peñas E., Frías J., Gómez R., Martinez-Villaluenga C. (2015). High-pressure improves enzymatic proteolysis and the release of peptides with angiotensin I converting enzyme inhibitory and antioxidant activities from lentil proteins. Food Chem..

[122] Zhu D., Guan D., Fan B., Sun Y., Wang F. (2022). Untargeted mass spectrometry-based metabolomics approach unveils molecular changes in heat-damaged and normal soybean. LWT.

[123] Manassero C.A., Vaudagna S.R., Sancho A.M., Añón M.C., Speroni F. (2016). Combined high hydrostatic pressure and thermal treatments fully inactivate trypsin inhibitors and lipoxygenase and improve protein solubility and physical stability of calcium-added soymilk. Innov. Food Sci. Emerg. Technol..

[124] Rane R., Marar T., Sonawane S.K., Dabade A. (2022). A review on Instant Controlled Pressure Drop Technology (DIC) associated with Drying technology and effect on quality characteristics. Food Chem. Adv..

[125] Pedrosa M.M., Cuadrado C., Burbano C., Allaf K., Haddad J., Gelencsér E., Takács K., Guillamón E., Muzquiz M. (2012). Effect of instant controlled pressure drop on the oligosaccharides, inositol phosphates, trypsin inhibitors and lectins contents of different legumes. Food Chem..

[126] Téllez-Pérez C., Alonzo-Macías M., Mounir S., Besombes C., Allaf T., Amami E., Allaf K. (2019). Instant Controlled Pressure-Drop DIC as a Strategic Technology for Different Types of Natural Functional Foods. Funct. Foods.

[127] Haddad J., Greiner R., Allaf K. (2007). Effect of instantaneous controlled pressure drop on the phytate content of lupin. LWT—Food Sci. Technol..

[128] Berk Z. (2017). Food extrusion. Engineering Foods for Bioactives Stability and Delivery.

[129] Nikmaram N., Leong S.Y., Koubaa M., Zhu Z., Barba F.J., Greiner R., Oey I., Roohinejad S. (2017). Effect of extrusion on the anti-nutritional factors of food products: An overview. Food Control.

[130] Singh S., Gamlath S., Wakeling L. (2007). Nutritional aspects of food extrusion: A review. Int. J. Food Sci. Technol..

[131] Shiferaw Terefe N., Buckow R., Versteeg C. (2015). Quality-related enzymes in plant-based products: Effects of novel food-processing technologies part 3: Ultrasonic processing. Crit. Rev. Food Sci. Nutr..

[132] Huang H., Kwok K.C., Liang H.H. (2008). Inhibitory activity and conformation changes of soybean trypsin inhibitors induced by ultrasound. Ultrason. Sonochem..

[133] Nadar S.S., Rathod V.K. (2017). Ultrasound assisted intensification of enzyme activity and its properties: A mini-review. World J. Microbiol. Biotechnol..

[134] Wu Y., Li W., Martin G.J., Ashokkumar M. (2021). Mechanism of low-frequency and high-frequency ultrasound-induced inactivation of soy trypsin inhibitors. Food Chem..

[135] Kumar R., Kumar A., Jayachandran L.E., Rao P.S. (2021). Sequential Microwave–Ultrasound assisted extraction of soymilk and optimization of extraction process. LWT.

[136] Zhu X., Wang W., Shen J., Xu X., Zhou G. (2018). Influence of gamma irradiation on porcine serum albumin structural properties and allergenicity. J. AOAC Int..

[137] Farag M.D.E.D.H. (1998). The nutritive value for chicks of full-fat soybeans irradiated at up to 60 kGy. Anim. Feed Sci. Technol..

[138] Taghinejad-Roudbaneh M., Ebrahimi S., Azizi S., Shawrang P. (2010). Effects of electron beam irradiation on chemical composition, antinutritional factors, ruminal degradation and in vitro protein digestibility of canola meal. Radiat. Phys. Chem..

[139] Ebrahimi-Mahmoudabad S., Taghinejad-Roudbaneh M. (2011). Investigation of electron beam irradiation effects on anti-nutritional factors, chemical composition and digestion kinetics of whole cottonseed, soybean and canola seeds. Radiat. Phys. Chem..

[140] Zhang Y., Wang S., Niu D., Huang Z., Gai L., Hang F. (2024). Effects of moderate dielectric barrier discharge (DBD) plasma treatment on the structure, antigenicity, and digestibility of casein. Food Hydrocoll..

[141] Dong S., Gao A., Zhao Y., Li Y., Chen Y. (2017). Characterization of physicochemical and structural properties of atmospheric cold plasma (ACP) modified zein. Food Bioprod. Process..

[142] Li Q., Shen F., He X., Xing C., Yan W., Fang Y., Hu Q. (2023). Modification of soy protein isolate using dielectric barrier discharge cold plasma assisted by modified atmosphere packaging. Food Chem..

[143] Liu Z.-W., Niu D., Zhou Y.-X., Cheng J.-H., Bekhit A.E.-D., Aadil R.M. (2021). Oxidation induced by dielectric-barrier discharge (dbd) plasma treatment reduces soybean agglutinin activity. Food Chem..

[144] Boutureira O., Bernardes G.J. (2015). Advances in chemical protein modification. Chem. Rev..

[145] Wu Y., Sessa D.J. (1994). Conformation of bowman-birk inhibitor. J. Agric. Food Chem..

[146] Wolf R.B., Cavins J.F., Kleiman R., Black L.T. (1982). Effect of temperature on soybean seed constituents: Oil, protein, moisture, fatty acids, amino acids and sugars. J. Am. Oil Chem. Soc..

[147] Nasri M. (2017). Protein hydrolysates and biopeptides: Production, biological activities, and applications in foods and health benefits. A review. Adv. Food Nutr. Res..

[148] Ma Y. (2010). Deactivation of Soybean Agglutinin by Enzyme Hydrolysis and Identification of Active Peptides from Soy Proteins. Ph.D. Thesis.

[149] Sugawara M., Ito D., Yamamoto K., Akita M., Oguri S., Momonoki Y.S. (2007). Kunitz soybean trypsin inhibitor is modified at its C-terminus by novel soybean thiol protease (protease T1). Plant Prod. Sci..

[150] EI-Gawad A.I., Hefny A.A., EI-Sayed E.M., Saleh F.A. (2015). Preparation Technique of Soymilk-Based Yoghurt and it’s Relation to Soybean Varieties and Anti-Nutritional Factors. J. Nutr. Food Sci..

[151] Qi N., Zhan X., Milmine J., Chang K., Li J. (2024). A novel thermophilic strain of Bacillus subtilis with antimicrobial activity and its potential application in solid-state fermentation of soybean meal. Microbiol. Spectr..

[152] Silva Júnior S., Tavano O., Demonte A., Rossi E., Pinto R. (2012). Nutritional evaluation of soy yoghurt in comparison to soymilk and commercial milk yoghurt. Effect of fermentation on soy protein. Acta Aliment..

