# Supplementary material for: Advancements in Inactivation of Soybean Trypsin Inhibitors
Source: Foods. 2025 Mar 12;14(6):975. doi: 10.3390/foods14060975 (PMC11941488; doi:10.3390/foods14060975)
Supplement: Supplementary file 1 [file foods-14-00975-s001.zip › foods-3508335-supplementary.pdf]

Supplementary Materials

Table S1. Various antinutritional factors and their harmful effects on the human body.

Name	Damage	Picture
Isoflavones	Isoflavones have phytoestrogenic activity and can bind to human estrogen receptors and interfere with the endocrine system.	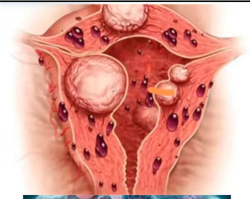
	Low-dose isoflavones may have anti-cancer effects, but excessive intake may promote estrogen-sensitive tumors.	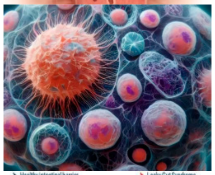
Soybean agglutinin	Soybean agglutinin can damage the tight junction of intestinal epithelial cells by binding to the glycoprotein, leading to increased intestinal permeability allowing undigested food particles, toxins, and bacteria to enter the blood, triggering systemic inflammation and autoimmune reactions.	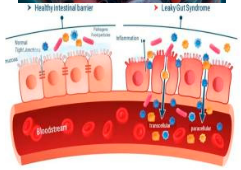
Lipoxygenase	Lipoxygenase catalyzed fatty acid oxidation products may further lead to rancidity, produce bad smell, affect the palatability.	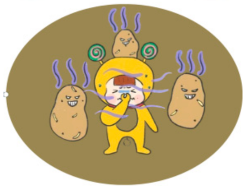
Saponins	Saponins bind to cholesterol on the cell membrane, change the permeability of the cell membrane, and then trigger an inflammatory response, leading to gastrointestinal discomfort.	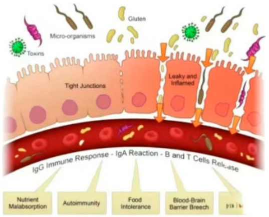
Trypsin inhibitors	Trypsin inhibitors may damage the gastrointestinal mucosa, leading to gastrointestinal bleeding, and indirectly cause liver damage. These toxic effects are particularly significant in the case of long-term intake of high doses of trypsin inhibitors.	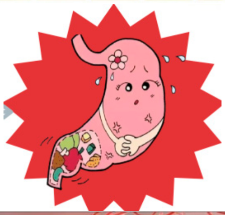
Urease	High urease activity may lead to oxidative stress and generate a large amount of reactive oxygen species (ROS), which may cause endothelial cell damage and inflammatory response. This inflammatory response may exacerbate the risk of atherosclerosis and cardiovascular disease.	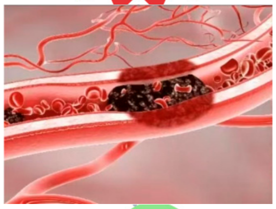
Phytic acid	Phytic acid is a strong complexing agent, which can combine with Ca, Mg, Fe, and Zn to form an insoluble complex and reduce the bioavailability of these mineral substances. Long-term ingestion of foods high in phytic acid may lead to mineral substance deficiency, anemia, osteoporosis, and so on.	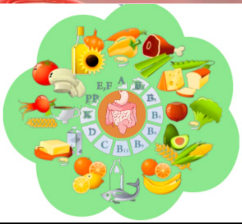

Antithiamins	Antithiamins cause thiamine deficiency by destroying the structure of thiamine or inhibiting its activity. Thiamin deficiency may cause beriberi, which manifests as nervous system abnormalities, cardio-vascular disorders , and metabolic disorders .	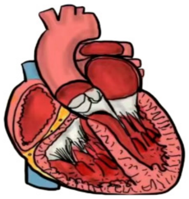
Tannic acid	Tannic acid has astringent properties, and excessive intake may irritate the gastrointestinal tract, causing diarrhea, stomach pain, and other symptoms of indigestion. Long-term intake may also cause gastroenteritis or liver damage.	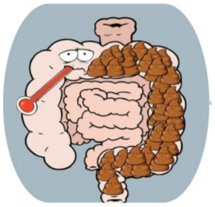
